# From Structural Kinematics to Thermomechanical Degradation in Polymer and Hybrid Negative Thermal Expansion Metamaterials

**DOI:** 10.3390/polym18121431

**Published:** 2026-06-08

**Authors:** Benjamín Méndez, Rodrigo Valle, César Garrido, Laurent Duchêne, Víctor Tuninetti

**Affiliations:** 1 Department of Mechanical Engineering, Universidad de La Frontera, Temuco 4811230, Chile; b.mendez02@ufromail.cl; 2 Construction Multidisciplinary Research Group, Facultad de Arquitectura, Construcción y Medio Ambiente, Universidad Autónoma de Chile, Talca 3460000, Chile; rodrigo.valle@uautonoma.cl; 3 Department of Mechanical Engineering, Universidad del Bío-Bío, Concepción 4081112, Chile; cgarrido@ubiobio.cl; 4 ArGEnCo Department, MSM Team, University of Liège, Quartier Polytech 1, Allée de la Découverte 9, 4000 Liège, Belgium; l.duchene@uliege.be

**Keywords:** negative thermal expansion (NTE), metamaterials, polymer additive manufacturing, thermoviscoelasticity, interfacial degradation, cohesive zone modeling (CZM), structural lattices, thermal fatigue, multi-material hybrids, glass transition temperature (Tg)

## Abstract

Metamaterials with tailored structural architectures enable negative thermal expansion through geometric mechanisms that counteract constituent-level positive expansion. This study evaluates the thermomechanical performance and structural limits of polymer and hybrid NTE lattices. We systematically classify the dominant kinematic mechanisms, including bimetallic bending, rotational squares, and re-entrant honeycombs, and quantify the inherent trade-offs between effective thermal contraction, structural stiffness, and mass efficiency. The analysis demonstrates that reliance on idealized linear–elastic and rigid-lever models leads to significant predictive discrepancies when evaluating the physical response of polymeric and hybrid prototypes. We establish that these deviations are fundamentally governed by localized stress singularities at multi-material interfaces and the profound thermoviscoelastic softening of polymers as they approach the glass transition temperature ($T_{g}$). We conclude that accurate prediction of the cyclic lifespan and dimensional stability of these systems requires a transition to coupled multiphysics frameworks. Specifically, integrating temperature-dependent cohesive zone modeling and time–temperature superposition principles is essential for capturing interfacial delamination and thermal ratcheting in high-performance polymeric NTE metamaterials.

## 1. Introduction

Controlling thermal expansion has become a fundamental requirement in advanced engineering systems, particularly in applications where dimensional stability directly affects performance, such as in aerospace structures, precision optics, microelectronic devices, and thermal management systems [1,2]. Conventional materials typically exhibit positive coefficients of thermal expansion (CTE), which can lead to thermal stresses, misalignment, fatigue, and long-term degradation in fluctuating thermal environments [3]. As engineering systems evolve toward greater precision and multifunctionality, the ability to control thermal expansion behavior has become a key design challenge [4,5]. In this context, materials and structures with negative thermal expansion (NTE) have attracted significant interest due to their ability to contract when heated [6,7]. Intrinsic NTE has been observed in specific crystalline materials, such as ZrW_2_O_8_ [8], as well as in lattice structures governed by transverse vibrational modes. However, these materials are often limited by their brittleness, narrow operating temperature ranges, and limited structural applicability, which restricts their integration into engineering systems [9].

To overcome these limitations, recent research has focused on metamaterials with defined architectures, where negative thermal expansion (NTE) arises from the geometric design rather than from the material’s intrinsic properties [10,11]. In these systems, local positive thermal expansion is transformed into global contraction through kinematic mechanisms such as bending, rotation, and articulation [12]. This paradigm has enabled the development of lattice-based metamaterials incorporating bimetallic elements, rotational units, re-entrant geometries, and hybrid architectures with highly tunable thermal responses [13,14,15]. It is worth noting that these systems can achieve effective thermal expansion coefficient (TEC) values that significantly exceed those of intrinsic materials, offering unprecedented control over thermomechanical behavior [16].

The rapid transition of NTE metamaterials from theoretical kinematics to physical prototypes has been fundamentally driven by advancements in Additive Manufacturing (AM) technologies [17,18]. Traditional subtractive or formative manufacturing paradigms are inherently unsuited for the fabrication of the complex, space-filling, and internal re-entrant geometries required to achieve macroscopic thermal contraction. AM techniques—particularly Laser Powder Bed Fusion (LPBF) for high-stiffness metallic lattices, Stereolithography (SLA) for high-resolution polymer networks, and multi-material Fused Filament Fabrication (FFF)—have provided unprecedented design freedom [19,20,21,22,23,24]. This fabrication versatility not only allows for the precise realization of intricate hinging and bending mechanisms at the microscale but also enables the seamless integration of dissimilar materials required for hybrid, thermally mismatched NTE architectures [25,26]. Consequently, the synergy between AM capabilities and topological design has become the indispensable cornerstone of modern mechanical metamaterial development. Furthermore, emerging approaches such as 4D printing and shape-changing materials have introduced adaptive thermal responses, expanding the functionality of NTE metamaterials toward intelligent and responsive systems [27,28]. These advancements have opened up new opportunities in applications ranging from thermal compensation components to adaptive structures and multifunctional materials.

Despite these advances, several significant challenges remain. First, the literature remains fragmented, with many studies focusing on specific geometries or isolated mechanisms, making it difficult to establish unified design principles or compare performance across different architectures [18]. Second, while analytical and numerical models have demonstrated strong and generally reliable predictive capabilities, discrepancies between simulations and experimental results persistently arise [29,30]. This gap is primarily attributed to manufacturing imperfections (e.g., micro-porosity and surface roughness), material heterogeneity, and interfacial stress concentrations in multi-material systems [19,21]. Furthermore, recent studies have shown that even microscopic geometric deviations can significantly alter thermomechanical behavior, particularly in systems dominated by bending [31]. Another critical limitation is the lack of systematic research on long-term performance and durability. Most existing studies focus on single cycles or short-term thermal loads, neglecting thermal fatigue, cyclic degradation, and viscoelastic creep, which are crucial for real-world implementation [32,33]. Consequently, the inherent trade-offs between thermal expansion, stiffness, energy absorption, and manufacturability remain unresolved, limiting the practical deployment of these materials.

Recent advances in data-driven design and machine learning have opened up new possibilities for exploring complex, high-dimensional design spaces, enabling the rapid inverse design of NTE metamaterials [32,34]. However, these approaches still face challenges related to data availability, model interpretability, and integration with physics-based methods [35]. Although several review articles have addressed different aspects of NTE metamaterials, including geometric design, auxetic behavior, and fabrication strategies [36], a comprehensive engineering-oriented framework that integrates geometry, performance, manufacturability and modeling approaches is still lacking [37]. In particular, there is a clear need to bridge the gap between conceptual design and practical implementation by establishing design guidelines that account for real-world constraints.

To address these challenges, this review presents a comprehensive and integrated analysis of NTE metamaterials from an engineering perspective. Unlike previous work, this study combines geometric classification, fabrication strategies, and modeling approaches within a unified framework, with a particular emphasis on design trade-offs and application-oriented selection criteria. Specifically, this work aims to: (i) classify NTE mechanisms according to their underlying kinematics; (ii) compare representative architectures in terms of thermal response, stiffness, and manufacturability; (iii) analyze fabrication strategies with an emphasis on polymer-based and multi-material systems; and (iv) review modeling approaches ranging from analytical formulations to data-driven design. Furthermore, this work identifies critical research gaps related to scalability, durability, and predictive modeling, providing a path toward the practical implementation of NTE metamaterials in engineering applications.

## 2. Mechanisms of Negative Thermal Expansion

Negative thermal expansion (NTE) in metamaterials arises from geometric mechanisms that transform local expansion into global contraction. These mechanisms are fundamentally different from the intrinsic negative thermal expansion observed in certain crystalline materials; instead, they are governed by structural kinematics and thermomechanical coupling.

The geometric design of the unit cells plays a fundamental role in determining the effective thermal and mechanical behavior of NTE metamaterials. Unlike conventional materials, where thermal expansion is an intrinsic property, metamaterials with specific architectures derive their response from geometric mechanisms that amplify or reverse local deformation. Over the past decade, various geometric configurations have been proposed, each with distinct advantages in terms of achievable NTE, structural stiffness, manufacturability, and functional applicability. However, these geometries are often analyzed in isolation, making it difficult to establish clear design guidelines. To address this limitation, the most representative architectures are classified and compared based on their underlying mechanisms and engineering performance.

To provide a unified perspective on these mechanisms, Table 1 presents a classification of representative NTE metamaterials based on their dominant deformation modes, including bimetallic bending, rotational kinematics, and flexible re-entrant behavior. This classification highlights the fundamental relationships between geometry, deformation mechanism, and macroscopic thermal response, allowing for a clearer comparison of the underlying design principles. By organizing the diverse range of reported architectures within a common framework, the table facilitates a systematic understanding of how different structural strategies translate into distinct thermomechanical behaviors.

**Figure 1:**
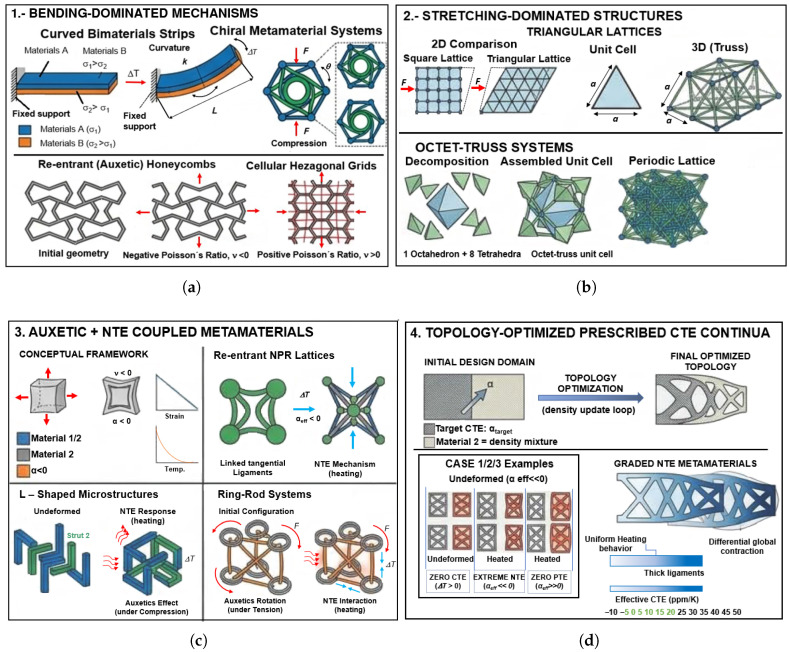
(**a**) Bending-dominated Mechanisms [38,39,40], (**b**) Stretching-dominated Structures [39,41], (**c**) Auxetic + NTE Coupled Metamaterials [42], (**d**) Topology-Optimized Prescribed CTE Continua [43,44].

Figure 1 illustrates the range of geometries associated with Table 1, providing a visual framework to facilitate the interpretation and classification of the geometric typologies considered within the scope of this research. In particular, the figure highlights configurations related to a negative Poisson’s ratio (NPR), also known as auxetic behavior, in which a structure expands laterally under tensile loading and contracts laterally under compression. This counterintuitive response is mainly governed by the architecture of the unit cell and allows for the design of structures with enhanced mechanical attributes, including improved energy absorption, indentation resistance, deformation adaptability, and tunable stiffness. Critically for this review, these same auxetic unit cell kinematics (e.g., rib unfolding or nodal hinging) often provide the foundational deformation mechanisms required to simultaneously achieve negative thermal expansion (NTE) when subjected to temperature gradients. Accordingly, the geometries shown in Figure 1 contribute to a clearer understanding of the novel and promising structural concepts currently explored in research on architected lattices and mechanical metamaterials.

### 2.1. Bimetallic Lattice Architectures

Bimetallic geometries, particularly the inverted trapezoidal lattice (ITL), represent one of the most effective strategies for achieving high negative thermal expansion. These structures rely on the differential thermal expansion between the joined materials, which induces bending and produces macroscopic contraction through geometric amplification [13,45]. Among all the configurations described, the ITL exhibits some of the highest negative thermal expansion values, reaching coefficients as low as $-74.4\times 10^{-6}$ K^−1^ [13]. This performance is attributed to the composite rod mechanism, which transforms local curvature into directional contraction and allows for strong amplification of thermal effects. From an engineering perspective, these structures offer exceptional tunability through geometric parameters such as element length ratios, inclination angles, and material combinations. However, their reliance on multi-material interfaces introduces significant challenges related to interfacial stresses, manufacturing complexity, and long-term durability under cyclic thermal loads [35].

### 2.2. Rotational and Stretching-Dominated Architectures

Rotational unit systems, including triangular lattices connected by vertices and struts, represent a class of metamaterials in which thermal expansion induces the coordinated rotation of rigid elements [12,14]. These mechanisms typically result in isotropic or quasi-isotropic negative thermal expansion (NTE) behavior, making them suitable for applications requiring uniform dimensional stability. Unlike systems dominated by bending, architectures dominated by tension exhibit higher specific stiffness and better load-bearing capacity, as predicted by cellular solid theory [39]. As a result, triangular configurations provide an optimal balance between thermal performance and mechanical robustness [14]. However, their achievable NTE magnitude is generally lower than that of bimetallic systems, highlighting a fundamental trade-off between stiffness and thermal response.

### 2.3. Flexible and Re-Entrant Architectures

Flexible structures, such as re-entrant honeycombs, achieve negative thermal expansion (NTE) through the bending and articulation of flexible elements, whereby local expansion is transformed into global contraction [38,46]. These structures are characterized by their ability to withstand large deformations and exhibit greater energy absorption capacity, making them attractive for applications involving impact mitigation and adaptive systems [47]. One of their main advantages lies in their simplicity and ease of fabrication, particularly when manufactured using polymer-based additive manufacturing techniques [15]. However, the inherently low stiffness of these systems limits their use in structural applications where dimensional stability must be maintained under mechanical loads.

### 2.4. Mechanisms of Parametric and Bio-Inspired Architectures

Recent advances have introduced more complex geometries based on parametric and bio-inspired design principles. Examples include networks based on Bézier curves and structures inspired by Islamic geometries, which allow for continuous control of deformation trajectories and greater adjustability of thermal response [4,15]. Unlike conventional lattice geometries, their response is not limited to a fixed contraction mechanism but can be programmed to exhibit positive or negative thermal expansion, depending on the configuration of the unit cell. In particular, it has been demonstrated that four-fold symmetric patterns, composed of articulated elements, can achieve adjustable thermal responses by varying the hinge angles, stiffness, and connectivity. Zhang et al. [15] demonstrated that such systems can transition between positive and negative effective coefficients of thermal expansion (CTE), highlighting the potential of geometry-driven programmability in thermal metamaterials.

### 2.5. Comparative Analysis and Design Guidelines

Table 2 summarizes representative geometries of NTE metamaterials, highlighting their underlying mechanisms, achievable thermal expansion, mechanical performance, and ease of fabrication. This comparison provides a concise overview of the key advantages and disadvantages that influence design selection [48,49].

### 2.6. Design Trade-Offs and Selection Criteria

The comparative analysis reveals that the selection of an appropriate NTE architecture should be guided by the specific requirements of the application, rather than by maximizing a single performance metric; for example, bimetallic networks are ideal for applications requiring high thermal contraction, but their susceptibility to thermal fatigue, interfacial degradation, and manufacturing limitations must be carefully considered [35]. On the other hand, rotational architectures provide greater mechanical stability and isotropy, making them more suitable for load-bearing structures [14]. Similarly, re-entrant and flexible systems are advantageous in applications requiring energy absorption and high deformation capacity [38], while parametric and bio-inspired designs offer greater adaptability at the cost of increased complexity [4]. These observations underscore the need for integrated design methodologies that simultaneously account for thermal, mechanical, and manufacturing constraints. In this context, multi-objective optimization frameworks and data-driven approaches are emerging as key tools for navigating the complex design space of NTE metamaterials [34].

## 3. Engineering Classification of Architectures

The performance of negative thermal expansion (NTE) metamaterials is fundamentally determined by the geometric configuration of their unit cells, which dictates how local thermal expansion is transformed into macroscopic contraction. Unlike conventional classifications based solely on topology, an engineering-oriented perspective requires evaluating these architectures in terms of their effective thermal response, mechanical stiffness, manufacturability, and application-specific functionality. Over the past decade, a wide range of geometries has been proposed, including bimetallic lattices, rotational systems, flexible re-entrant structures, and advanced parametric designs. Each of these leverages distinct deformation mechanisms, such as bending, articulation, or coordinated rotation, to achieve NTE behavior. As discussed in the literature, these architectures exhibit markedly different performance ranges, with thermal expansion coefficients varying from moderate to highly negative values, depending on geometric parameters and material combinations [4,13,14,15].

However, these designs are often analyzed in isolation, making it difficult to establish clear selection criteria for engineering applications. To address this limitation, this section provides a systematic classification of the most relevant NTE architectures, emphasizing their underlying mechanisms and, above all, their advantages and disadvantages in terms of performance and their practical implications for design.

### 3.1. Bimetallic Architectures

The inverted trapezoidal lattice (ITL) represents one of the most effective bimetallic architectures for achieving pronounced negative thermal expansion (NTE). This configuration consists of bars made of two materials arranged in an inverted trapezoidal geometry, where the differential thermal expansion between the constituent materials induces bending and rotation of the inclined elements. As the temperature increases, this coordinated kinematic response produces a net macroscopic contraction, generally in the vertical direction.

**Figure 2:**
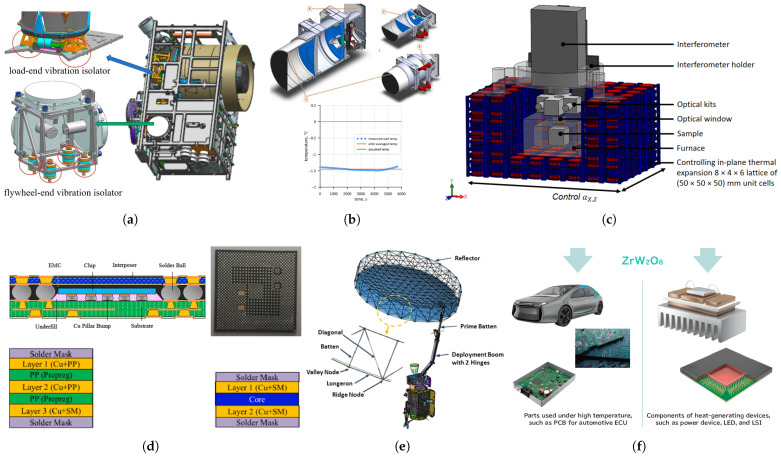
Critical engineering factors and architectural solutions enabled by negative thermal expansion (NTE) materials across multi-scale systems: (**a**) High-resolution CubeSat platform requiring micro-vibration suppression and strict dimensional stability to ensure optical performance [67]; (**b**) Passive thermal control systems in space applications, where heat transfer is dominated by conduction and radiation (key components shown: **1.** optical baffle, **2.** telescope housing, **3.** detector backplane, and **4.** mounting interfaces), highlighting the need for materials with tunable thermal properties [68]; (**c**) Low thermal expansion structural frameworks for precision engineering and metrology, where lattice-based architectures can minimize thermally induced deformation [69]; (**d**) Inverted trapezoidal lattice (ITL) unit cell based on a bi-material rod mechanism, enabling tailored negative thermal expansion behavior through geometry–material coupling [13]; (**e**) Advanced symmetric lattice architectures designed to achieve isotropic thermal response and enhanced structural stability under thermal loading [70]; (**f**) Negative Thermal Expansion Material (ZrW_2_O_8_) [71].

Luo et al. [13] demonstrated that ITL structures can achieve effective thermal expansion coefficients as low as −74.4 × 10^−6^ K^−1^ over a temperature range of 20 °C to 200 °C, outperforming equivalent triangular configurations by a factor of more than 2.5 [13]. This improved performance arises from a geometric amplification mechanism analogous to internal lever systems in multistable metamaterials [65,66], where local curvature is transformed into global contraction. At the heart of this behavior lies the concept of a bimetallic Composite Bar, which exhibits effective internal contraction within a so-called virtual bar (Figure 2a). The effective thermal expansion coefficient of this composite element can be expressed as

(1) $$\bar{\alpha }=\frac{\alpha_{b}r-\left(\alpha_{b}-\alpha_{a}\right)L}{r}$$

where $\alpha_{a}$ and $\alpha_{b}$ are the thermal expansion coefficients of the constituent materials, and L and r define the geometric proportions of the composite system. This internal contraction mechanism is subsequently amplified at the network level through the rotation of the inclined elements forming the trapezoidal unit cell (Figure 2b), resulting in a pronounced macroscopic NTE response.

The performance of ITL structures is strongly influenced by key geometric and material parameters. Parametric studies have shown that the L/r ratio plays a fundamental role in controlling the magnitude of contraction, with higher values leading to greater negative thermal expansion (NTE) at the expense of increased internal stresses [13]. Similarly, the contrast between the thermal expansion coefficients of the constituent materials directly influences the induced curvature and rotational response. Furthermore, the relative cell height and inclination angles affect the degree of rotational amplification, where more compact geometries tend to increase the magnitude of NTE but may introduce instability phenomena such as local buckling or manufacturing difficulties [18,25,72].

Based on this concept of a composite rod, Luo et al. (2019) [13] extended the mechanism to periodic lattice configurations by arranging multiple bi-material elements within a symmetric unit cell (Figure 2b). Under thermal loading, each unit cell undergoes coordinated deformation in which the contraction of the individual virtual rods results in an overall reduction of the structure. This architecture allows for precise control of anisotropic thermal behavior through geometric design and material selection, highlighting the potential of ITL systems as a platform for tunable, high-performance NTE metamaterials.

Beyond the specific case of inverted trapezoidal lattices, the relationship between the unit cell architecture and macroscopic thermomechanical behavior has been systematically investigated in bimetallic metamaterials. Chen et al. [14] analyzed a family of six bimetal-based lattice configurations, classified into vertex-connected (VT, VS, VH) and strut-connected (ST, SS, SH) systems, revealing that geometric connectivity plays a fundamental role in determining thermal and mechanical responses. Their results demonstrate that the average vertex angle (θ) and the type of connectivity not only define the achievable range of effective thermal expansion, but also significantly influence stiffness and relative density. In particular, a direct coupling emerges between normalized thermal expansion ($\bar{\alpha }$) and specific stiffness ($\bar{E}=E^{*}/\bar{\rho }$), highlighting an intrinsic trade-off in the design of bimetallic networks. For example, vertex-connected configurations, such as VS and VH, exhibit high specific stiffness combined with near-zero or moderately negative thermal expansion, making them ideal for applications that require dimensional stability under mechanical load. In contrast, strut-connected systems, such as SH, can achieve greater thermal expansion responses, both positive and negative, while maintaining relatively high stiffness, which is advantageous for thermally activated or transformable structures [14].

These findings reinforce the idea that, beyond material selection, the topology and connectivity of the unit cell are critical design variables that allow for the tuning of coupled thermomechanical properties, providing a broader design space for the engineering of high-performance NTE metamaterials.

### 3.2. Rotational/Triangular Systems

Rotational and triangular lattice systems represent a class of metamaterials with negative thermal expansion (NTE) in which macroscopic contraction arises from coordinated kinematic motion, rather than from material incompatibility. In these architectures, rigid or semi-rigid units—such as triangles, squares, or polygons—are connected by flexible hinges or elastic joints, allowing rotational degrees of freedom to dominate the structural response. When subjected to thermal loading, the local expansion of the elements induces a controlled rotation of the units, resulting in an overall contraction of the network [12,14].

Unlike bimetallic systems, where the NTE is due to differential thermal expansion between the constituent materials, rotational architectures rely primarily on geometric reconfiguration. This mechanism allows for the design of structures using single-material or homogeneous compositions, which simplifies fabrication and reduces problems associated with interfacial stresses [12].

On the other hand, triangular configurations, in particular, have been extensively studied due to their favorable mechanical behavior. According to cellular solid theory, these systems are typically tension-dominated, which provides higher specific stiffness and better load-bearing capacity compared to bending-dominated architectures [39]. As a result, triangular and vertex-connected networks offer an optimal balance between mechanical robustness and thermal functionality, although their achievable negative thermal expansion (NTE) is generally lower than that of bimetallic systems [14].

Rotational systems, such as rotating squares, further extend this concept by enabling quasi-isotropic thermal contraction through symmetric kinematic motion. These configurations exhibit predictable deformation trajectories, governed by the geometry and connectivity of the hinges, making them suitable for applications requiring uniform dimensional stability [12]. However, their behavior is highly sensitive to hinge stiffness and geometric accuracy, which can significantly influence the effective thermal response.

In general, rotational and triangular architectures offer a robust and manufacturable alternative to bimetallic systems, particularly in applications where structural integrity and isotropy are prioritized over extreme negative thermal expansion (NTE) performance. Their behavior highlights the fundamental role of kinematic design in controlling thermomechanical properties, regardless of material composition.

### 3.3. Flexible/Re-Entrant

Flexible and recessed architectures constitute a class of metamaterials with negative thermal expansion (NTE) in which thermal contraction is achieved through the deformation of flexible structural elements, rather than through the movement of a rigid body or material mismatch. These systems typically incorporate inclined or re-entrant cellular geometries with flexible joints, allowing for large kinematic transformations under thermal loading. As the temperature increases, local expansion of the material induces bending and inward deformation of the cellular walls, resulting in macroscopic contraction of the structure [38,46].

Recessed honeycomb structures represent one of the most studied configurations within this category. Characterized by inward-sloping cellular ribs, these geometries exhibit auxetic behavior (negative Poisson’s ratio), closely related to their ability to produce negative thermal expansion. The coupling between lateral contraction and axial deformation allows for an efficient transformation of local expansion into global contraction, particularly in regimes dominated by bending [38,46].

From a mechanical standpoint, flexible architectures differ significantly from systems dominated by tension, such as triangular trusses. Their deformation is governed primarily by bending and hinge motion, which allows for a high capacity to accommodate deformation and greater energy absorption [47]. As a result, these systems are particularly attractive for applications involving impact mitigation, adaptive structures, and transformation systems. Furthermore, their relatively simple geometry and compatibility with polymer-based additive manufacturing techniques facilitate their fabrication at multiple scales [15]. However, this increased deformability comes at the expense of lower structural stiffness and load-bearing capacity. Compared to bimetallic and rotational architectures, re-entrant systems typically exhibit lower specific stiffness and greater sensitivity to geometric imperfections, which can significantly affect their thermomechanical response [49]. Furthermore, their performance is strongly influenced by manufacturing accuracy, especially in the definition of hinge regions and thin elements.

However, a critical limitation in these polymer-based flexible architectures is their inherent viscoelasticity, which introduces severe time- and temperature-dependent nonlinearities. From a macromolecular perspective, as the operational temperature approaches the glass transition temperature ($T_{g}$), the polymer chains acquire sufficient thermal energy to overcome secondary intermolecular forces, initiating long-range segmental motion [73]. This thermomechanical transition manifests macroscopically as a precipitous drop in the storage modulus ($E^{\prime }$), often by two to three orders of magnitude, accompanied by a sharp peak in the loss tangent (tanδ). In the context of NTE metamaterials, this phase transition fundamentally violates the assumption of rigid-lever kinematics upon which theoretical geometric contraction models are based [15].

In bending-dominated NTE mechanisms, the transformation of local positive thermal expansion into macroscopic contraction requires the constituent struts and hinges to efficiently store and transfer elastic strain energy [49]. When operating near $T_{g}$, this elastic energy is instead largely dissipated through viscous flow. Rather than driving coordinated network contraction, the thermally induced local stresses cause the softened polymeric hinges to undergo pronounced stress relaxation and viscoplastic creep [36]. Consequently, the effective thermal expansion coefficient ceases to be a purely geometric constant and devolves into a transient, load-history-dependent function, $\alpha_{eff}\left(t,T\right)$. Under cyclic thermal loading, this viscous dissipation prevents the complete recovery of the lattice to its original configuration, leading to progressive strain accumulation (thermal ratcheting) and severe structural hysteresis. This establishes a rigid design boundary: the functional temperature envelope of polymeric NTE systems is strictly constrained by the viscoelastic relaxation spectrum of the base material, necessitating advanced time–temperature superposition modeling to accurately predict long-term dimensional stability [18].

In general, flexible and recessed architectures constitute an effective strategy for achieving moderate levels of negative thermal expansion (NTE), while offering advantages in terms of deformability, ease of fabrication, and multifunctionality. Their behavior highlights the role of flexible mechanisms in enabling thermally induced deformation, expanding the design space of metamaterials beyond rigid or multi-material systems.

### 3.4. Advanced Parametric and Bio-Inspired Architectures

**Figure 3:**
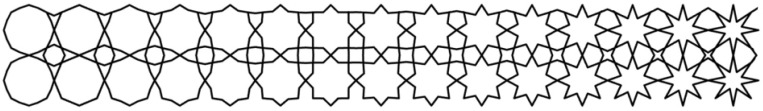
Architectural inspiration based on Islamic geometric patterns, exemplified by the dome of the Friday Mosque in Saveh, Iran. Reproduced from [74].

From an engineering and performance perspective, modular and bio-inspired architectures represent an emerging class of highly adaptable NTE metamaterials. Figure 3 shows representative examples of these geometries and their geometric inspiration, which leverage the principles of symmetry and tessellation. These bio-inspired configurations benefit from a more uniform stress distribution compared to beam-based lattice structures, which reduces stress concentrations and enables smoother kinematic transitions. Furthermore, their parametric nature allows for integration with computational design tools, such as topological optimization and machine learning, which facilitates the exploration of large design spaces and enables inverse design strategies [34]. Consequently, they offer a promising path toward programmable thermal behavior and multifunctional performance.

Despite these advantages, their practical implementation remains a challenge due to high manufacturing complexity and performance reliability. Their sensitivity to geometric imperfections, hinge flexibility, and manufacturing tolerances can significantly influence the resulting thermomechanical response. Furthermore, their implementation often requires advanced manufacturing techniques, particularly in the context of multi-material or high-resolution additive manufacturing [74]. Ultimately, their development represents a shift from discrete, geometry-based mechanisms toward continuous and highly adaptable systems, opening new opportunities for smart materials and adaptive engineering applications.

## 4. Design Trade-Offs and Selection Framework

### 4.1. Stiffness–Energy Absorption Trade-Off

**Figure 4:**
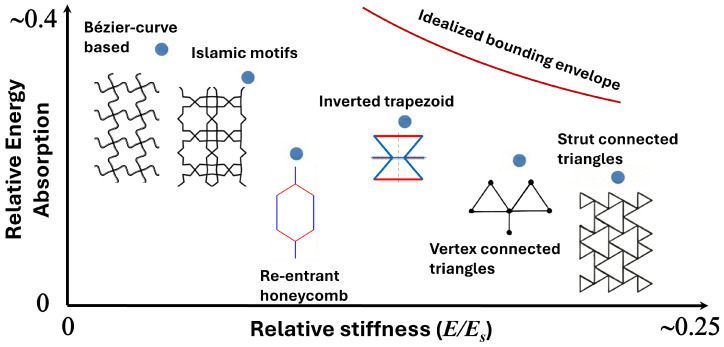
Stiffness–energy absorption map of NTE metamaterials, illustrating the trade-off between normalized stiffness ($E/E_{s}$) and relative energy absorption. The plotted regions and the ‘idealized bounding envelope’ represent theoretical approximations estimated for a constant relative density ($\rho^{*}/\rho_{s}\approx 15–20\%$), derived utilizing the scaling methodologies of Gibson and Ashby [39]. Actual performance of AM-fabricated lattices typically exhibits significant scatter (e.g., a 20–40% stiffness reduction) due to manufacturing variability.

To complement the qualitative comparison of NTE architectures presented in Table 2, a quantitative analysis was conducted to evaluate the mechanical performance of representative geometries. The resulting stiffness–energy absorption map is shown in Figure 4. Rather than plotting discrete experimental data points with inherent scatter, this map provides an idealized bounding envelope constructed theoretically by assuming a constant relative density range ($\rho^{*}/\rho_{s}\approx 0.15–0.20$). The performance regions were estimated utilizing the well-established cellular solids scaling laws from Gibson and Ashby [39] alongside the analytical methodologies for chiral and re-entrant systems formulated by Prall and Lakes [47].

The horizontal axis corresponds to normalized stiffness ($E/E_{s}$), capping at a theoretical upper limit of ∼0.25 for this density range, which represents the effective elastic modulus relative to the base material. The vertical axis quantifies the relative energy absorption (reaching up to ∼0.4), associated with deformation mechanisms such as bending, buckling, and rotation of the cellular wall. It is critical to note that for additively manufactured lattices, inherent geometric variability and process-induced micro-porosity can shift the effective stiffness downwards by 20% to 40% compared to these idealized analytical predictions [21,33]. Therefore, these bounding regions must be treated as theoretical upper-limit performance targets requiring rigorous uncertainty quantification in practical structural applications. As illustrated in Figure 4, different classes of metamaterial architectures occupy distinct regions of the performance space.

Tensile-dominated architectures, including vertex-connected and strut-connected triangles, are located in the high-stiffness region of the map (with relative stiffness approaching ∼0.25). This reflects their superior load-bearing capacity and stretch-dominated structural efficiency [19]. In stark contrast, highly compliant chiral and parametric designs—such as Bézier-curve based networks and Islamic motifs—occupy the extreme low-stiffness, high-energy-absorption domain (reaching up to ∼0.4 relative energy absorption). Their deformation is governed by severe bending and rotational mechanisms, making them ideal for extreme damping and impact-absorbing applications [75].

Intermediate configurations, such as re-entrant honeycombs and inverted trapezoid geometries, lie in the central region of the map in Figure 4. These architectures exhibit a balanced performance profile, combining moderate structural stiffness with appreciable energy dissipation capabilities [38]. Furthermore, certain space-filling topologies, such as the Kelvin-type (truncated octahedron) lattice, have recently demonstrated exceptional energy dissipation efficiency that bridges these domains. These architectures leverage a sequential transition from elastic–plastic buckling to nodal shear rotation, enabling effective damping values ($\beta_{eff}$) that can exceed unity [76]. Unlike purely bending-dominated forms, the stretch-dominated load path in metallic Kelvin lattices preserves cyclic stability and concentrates plastic work at junction nodes, offering a superior balance between high-performance energy absorption and ductility [76].

Furthermore, the application of polymeric composites and re-entrant architectures is increasingly critical under extreme dynamic loading conditions. Recent computational perspectives have demonstrated the exceptional energy dissipation capabilities of high-strength carbon/epoxy polymer composites when utilized for blast and explosion resistance in structural armors, achieving nearly 30% improvements in energy absorption compared to monolithic designs [77]. Bridging these extreme-loading composite behaviors with the kinematic flexibility of NTE re-entrant lattices represents a highly promising frontier for developing multifunctional impact mitigation systems.

In general, the stiffness–energy absorption map shown in Figure 4 serves as a practical decision-making tool for selecting NTE metamaterial geometries based on specific performance requirements. Designs that prioritize dimensional stability and load-bearing capacity benefit from high-stiffness triangular configurations, while applications involving impact mitigation or massive deformations favor Bézier or rotational systems [66]. Hybrid, re-entrant, or parametric architectures provide additional flexibility by allowing for adjustable trade-offs between these conflicting properties [18].

### 4.2. Integrated Performance Insights and Design Criteria

**Figure 5:**
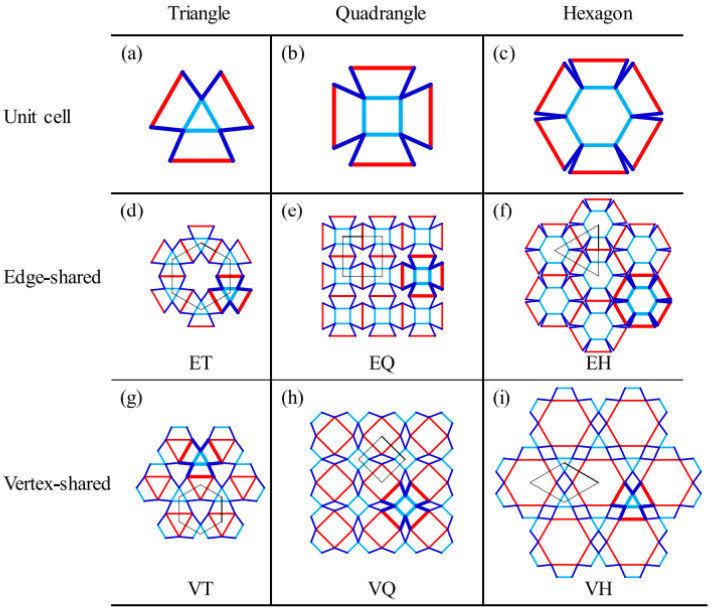
Representative unit cells and periodically arrayed metamaterials composed of trapezoid units. (**a**–**c**) Triangle, quadrangle, and hexagon representative unit cells composed of trapezoid elements. (**d**–**f**) Edge-shared periodic arrays constructed by sharing the long red beams: Edge-shared Triangle (ET), Edge-shared Quadrangle (EQ), and Edge-shared Hexagon (EH). (**g**–**i**) Vertex-shared periodic arrays constructed by sharing beam vertices: Vertex-shared Triangle (VT), Vertex-shared Quadrangle (VQ), and Vertex-shared Hexagon (VH). Reproduced from [78].

Furthermore, beyond the stiffness-energy relationship, the selection of NTE architectures must take into account additional coupled parameters, such as achievable thermal expansion, manufacturability, and anisotropy. Combined analysis of these factors reveals specific design guidelines for engineering applications. Figure 5 illustrates representative geometric configurations and connectivity strategies that influence these properties. Bimetallic systems, particularly the inverted trapezoidal lattice (ITL), achieve the highest reported NTE values due to their composite rod amplification mechanism [13]. This makes them highly suitable for applications requiring significant thermal compensation or actuation [8]. However, their reliance on multi-material interfaces poses challenges related to durability and manufacturing complexity.

In contrast, triangular and rotational architectures offer more balanced performance, combining moderate negative thermal expansion with high specific stiffness, making them ideal for load-bearing structures that require dimensional stability [14]. Flexible and re-entrant systems, while exhibiting lower stiffness, offer superior energy absorption and simplified manufacturing, particularly in polymer-based manufacturing contexts [38]. Manufacturing considerations also influence design selection. Architectures with simple geometries, such as re-entrant honeycombs, are better suited for large-scale production, while advanced parametric and bio-inspired designs, such as Bézier-based systems or Islamic geometric motifs, offer greater adaptability at the cost of increased manufacturing complexity [4,15]. As suggested by the connectivity variations shown in Figure 5, even subtle geometric modifications can significantly alter the system’s mechanical and thermal response.

Furthermore, the choice between isotropic and anisotropic thermal behavior is crucial. Rotational and triangular systems generally exhibit isotropic or quasi-isotropic responses, which is advantageous for uniform dimensional stability [12]. In contrast, anisotropic architectures, such as ITL and re-entrant systems, allow for directional control of thermal expansion, making them suitable for specialized applications where specific deformation is required [13,14]. In this way, these observations highlight that no single architecture simultaneously optimizes all performance parameters. Instead, the design of NTE metamaterials requires a multi-objective approach that balances thermal response, mechanical performance, and manufacturability, reinforcing the need for integrated design frameworks.

To support this multi-objective design approach, Table 3 summarizes the relationships between architectural concepts, fabrication strategies, and application domains for representative NTE metamaterials. By integrating geometric design, manufacturability considerations, and functional performance, this framework provides a practical guide for selecting appropriate configurations based on specific engineering requirements. In particular, it highlights how different structural classes are associated with distinct fabrication routes and application scenarios, facilitating the transition from conceptual design to real-world implementation.

## 5. Modeling Approaches

The mathematical prediction of negative thermal expansion (NTE) in metamaterials is based on a combination of analytical, numerical, and data-driven modeling approaches. These methods offer complementary capabilities at different stages of design, from rapid conceptual evaluation to high-fidelity validation. While analytical formulations capture the fundamental relationships between geometry and thermomechanical response, numerical and data-driven approaches enable accurate prediction in complex architectures and broad design spaces.

### 5.1. Analytical Modeling

Analytical models describe the effective thermal behavior of metamaterials using simplified kinematic relationships and beam theory approximations. In bimetallic systems, the curvature induced by differential thermal expansion can be described using Timoshenko-type formulations, where the difference in thermal expansion coefficients (CTE) generates a bending deformation that is subsequently amplified at the structural level [13]. For truss-based systems, the effective mechanical response is commonly described using Gibson–Ashby scaling laws:

(2) $$\frac{E^{*}}{E_{s}}=C_{1}{\left(\frac{\rho^{*}}{\rho_{s}}\right)}^{n_{1}}$$

where $E^{*}$ and $\rho^{*}$ represent the effective modulus and density of the network, while $E_{s}$ and $\rho_{s}$ correspond to the properties of the base material, and $C_{1}$ is a geometric proportionality constant. The exponent $n_{1}$ reflects the dominant deformation mechanism, distinguishing between architectures dominated by tension ($n_{1}\approx 1$) and those dominated by bending ($n_{1}\approx 2$) [39,79]. In rotational and re-entrant systems, geometric parameters such as strut length and inclination angle govern the effective coefficient of thermal expansion through kinematic constraints, allowing the transformation of local expansion into global contraction [12,38]. Although these analytical formulations are based on simplifying assumptions, they provide essential physical insight and allow for a rapid evaluation of design parameters, making them particularly valuable during the initial design stage.

### 5.2. Finite Element Modeling

Finite element analysis (FEA) is the primary numerical tool for evaluating thermomechanical coupling in metamaterials with negative thermal expansion (NTE). In this method, a representative unit cell is subjected to a prescribed temperature change ΔT, and the resulting displacement field is used to calculate the effective strain. The effective thermal expansion coefficient is then obtained as

(3) $$\alpha_{eff}=\frac{\varepsilon_{avg}}{\Delta T}$$

where $\varepsilon_{avg}$ is the average normal strain over the unit cell [35]. Finite element analysis (FEA) allows for the analysis of complex geometries, nonlinear deformation, and material heterogeneity, which are difficult to capture analytically. Its accuracy depends largely on mesh refinement, boundary conditions, and the inclusion of temperature-dependent material properties. Comparisons with experimental results have shown good agreement, with deviations typically less than 5% for well-calibrated models [13,14]. This validates FEA as a reliable tool for predicting effective thermomechanical behavior and guiding structural optimization.

However, while standard FEA adequately predicts the idealized kinematic response of homogeneous geometries, it fundamentally struggles with multi-material and hybrid architectures. A critical superficiality in current literature is the widespread reliance on continuous-mesh, perfectly bonded linear–elastic interfaces. In hybrid NTE metamaterials (e.g., polymer–metal or dissimilar alloy bimetallics), the extreme mismatch in the coefficients of thermal expansion (Δα) generates severe shear and peeling stress concentrations at the free edges of the interface. Under idealized linear–elastic FEA, these bi-material corners manifest as stress singularities, predicting infinite stress that fails to capture physical reality.

To accurately model the transition from theoretical kinematics to real-world thermomechanical degradation, numerical approaches must transition to fracture mechanics frameworks. Specifically, the implementation of Cohesive Zone Modeling (CZM) utilizing temperature-dependent Traction-Separation Laws (TSL) is highly recommended. While the direct application of CZM to multi-material NTE metamaterials remains a critical open research gap, there is strong methodological precedent for its transferability. Advanced fracture and damage mechanics are currently the established standard for predicting cyclic thermal fatigue and interfacial failure in analogous multi-material systems. For instance, temperature-dependent CZM is heavily utilized to simulate progressive delamination in Carbon Fiber Reinforced Polymer (CFRP) laminates [80], while advanced strain-energy damage models are required to predict thermal fatigue cracking at the dissimilar material interfaces of microelectronic solder joints [81]. CZM mitigates the singularity issue by defining a finite interfacial cohesive energy and a maximum traction criterion, allowing for the accurate simulation of damage initiation, micro-yielding, and progressive delamination under cyclic thermal fatigue. Without integrating these transferred multiphysics degradation models, computational predictions of high-performance NTE metamaterials remain purely theoretical and drastically overestimate the cyclic lifespan of the structures.

### 5.3. Data-Driven and Optimization Approaches

Recent advances in machine learning (ML) have revolutionized the design paradigms for NTE metamaterials, offering a powerful alternative to computationally expensive iterative modeling. Because experimental fabrication and testing of thousands of prototypes is economically unfeasible, data-driven frameworks overwhelmingly rely on high-throughput Finite Element Analysis (FEA) to generate massive training datasets. These datasets typically comprise $10^{4}$ to $10^{5}$ unit cell variations, mapping specific geometric parameters (e.g., strut thickness, hinge angles, curvature) to their effective thermomechanical responses [32]. When properly calibrated, surrogate ML models trained on these FEA datasets exhibit exceptional predictive fidelity, frequently achieving coefficient of determination ($R^{2}$) values exceeding 0.95 and near-zero Root Mean Square Errors (RMSE) on CTE predictions [34,82].

The selection of the ML architecture fundamentally dictates the model’s capability. Convolutional Neural Networks (CNNs) are extensively utilized for evaluating voxel-based or pixelated topological geometries, efficiently capturing spatial hierarchies to predict effective stiffness and CTE. Deep Autoencoders are frequently employed for dimensionality reduction, compressing highly complex 3D lattice topologies into low-dimensional latent spaces that are significantly easier to optimize. For the generation of entirely novel structural classes, Generative Adversarial Networks (GANs) have shown immense promise by learning the underlying distribution of high-performance metamaterials and synthesizing new topologies that do not exist in the training data [83]. Alternatively, when data is scarce or computationally expensive to generate, Gaussian Processes (GPs) are utilized alongside Bayesian Optimization to construct probabilistic surrogate models, allowing for the efficient exploration of the design space with rigorously quantified uncertainty boundaries [84].

Despite these algorithmic successes, a critical hurdle remains in the inverse design of NTE metamaterials: the “non-uniqueness” challenge. Inverse design seeks to input a target macroscopic property (e.g., a specific negative CTE) and output the optimal unit cell geometry. However, this is an inherently ill-posed problem because the structure–property mapping is non-bijective; multiple highly distinct topological geometries can yield the exact same effective CTE. If an ML model is trained naively on a single target property, it often fails to converge or outputs physically unmanufacturable geometries. Overcoming this non-uniqueness challenge requires the implementation of multiphysics, multi-objective loss functions. By simultaneously constraining the model to achieve the target CTE while maximizing normalized stiffness ($E/E_{s}$) and minimizing relative density ($\rho^{*}/\rho_{s}$), the dimensionality of the solution space is constrained, enabling the algorithm to converge on a unique, structurally robust, and physically manufacturable NTE architecture [18,83].

Topological and multi-objective optimization frameworks have also been applied to simultaneously balance conflicting requirements, such as maximizing the negative thermal expansion coefficient (NTE) while maintaining structural stiffness and minimizing density [18]. Despite their potential, these methods require extensive datasets and computational resources, and their predictions must be experimentally validated to ensure their reliability. Despite their efficiency, ML-based methods typically require large datasets and may lack physical interpretability. Therefore, they are most effective when combined with analytical and numerical models, forming a hybrid framework that balances accuracy, speed, and physical understanding.

### 5.4. Experimental Validation and Multiscale Challenges

A fundamental aspect of modeling is the validation of predictions through experimental testing. Techniques such as digital image correlation (DIC) and thermal cycling tests are commonly used to measure deformation and evaluate the effective coefficient of thermal expansion (CTE). However, a challenge persists in the gap between simulation and reality, where experimentally measured behavior differs from numerical predictions. This discrepancy is primarily attributed to manufacturing defects, such as porosity, surface roughness, and geometric inaccuracies, as well as material inconsistencies at interfaces [35]. In the context of polymer-based additive manufacturing, this discrepancy is primarily attributed to process-induced defects; for example, poor layer adhesion and void formation in Fused Filament Fabrication (FFF) or over-curing and anisotropic shrinkage in Stereolithography (SLA) can drastically alter the predicted effective thermal expansion coefficient. Furthermore, the temperature-dependent viscoelastic nature of polymers, particularly as they approach their glass transition temperature, introduces nonlinearities that idealized elastic models fail to capture. To bridge this gap, it is necessary to develop multiscale models that incorporate these polymer-specific manufacturing imperfections, elastomeric hinge behaviors, and viscoelastic relaxations, enabling more accurate predictions of performance under real-world conditions.

### 5.5. Thermomechanical Degradation in Polymer-Based NTE Systems

The operational stability of polymer-based NTE metamaterials is intrinsically bounded by the thermoviscoelastic nature of the constituent materials. In bending-dominated mechanisms, the transformation of local positive expansion into macroscopic contraction requires the constituent hinges to efficiently store and transfer elastic strain energy. However, as the operational temperature approaches the glass transition temperature ($T_{g}$), this elastic energy is instead dissipated through viscous flow. Table 4 maps the thermal envelopes of common additive manufacturing (AM) polymers, establishing the rigid design boundaries for reliable kinematic performance.

When operating near these thermal boundaries, the thermally induced local stresses cause the softened polymeric hinges to undergo pronounced stress relaxation and viscoplastic creep. To quantitatively predict this long-term dimensional degradation (thermal ratcheting), the application of Time–Temperature Superposition (TTS) methodology is strictly required. TTS relies on the principle that the viscoelastic behavior of polymers at high temperatures over short durations is mathematically equivalent to their behavior at lower temperatures over extended durations.

Utilizing the Williams–Landel–Ferry (WLF) equation, empirical shift factors ($a_{T}$) can be calculated:

(4) $$\log a_{T}=\frac{-C_{1}\left(T-T_{ref}\right)}{C_{2}+T-T_{ref}}$$

where $T_{ref}$ is the reference temperature, and $C_{1}$ and $C_{2}$ are material-specific empirical constants. This shift factor is then directly applied to construct a long-term relaxation master curve, transforming the time-dependent modulus E(t,T) into a shifted master function $E(t/a_{T},T_{ref})$. By integrating this master curve into finite element models, the effective thermal expansion coefficient ceases to be a static geometric constant and is accurately modeled as a transient, load-history-dependent function, $\alpha_{eff}\left(t,T\right)$. This paradigm shift from static kinematic modeling to dynamic, time–temperature-dependent lifespan prediction is essential for the real-world deployment of polymeric metamaterials.

## 6. Discussion, Research Gaps, and Future Directions

The development of metamaterials with a negative coefficient of thermal expansion (CTE) has shown significant progress in terms of geometric design and theoretical modeling; however, several critical challenges remain that limit their practical application. One of the most significant issues is the strong interdependence between geometry, manufacturing processes, and material behavior. While many designs achieve exceptional CTE performance under idealized conditions, their implementation in the real world is often limited by manufacturing constraints, particularly in multi-material systems where interfacial bonding and residual stresses play a crucial role [35].

In multi-material NTE lattices, the interfacial boundaries represent the primary sites for structural failure; however, the physical degradation mechanisms differ fundamentally between material classes. Rather than treating these interfaces as isolated phenomena, it is critical to recognize their shared vulnerability to thermally induced stress singularities while distinguishing their distinct failure modes. In metallic hybrid structures, severe thermal cycling predominantly precipitates brittle fracture at intermetallic transition zones or accelerates galvanic corrosion at the discrete material interface [26]. Conversely, in polymeric and composite metamaterials, the primary degradation pathway is governed by profound mismatches in the constituent glass transition temperatures ($T_{g}$) and the onset of viscoelastic creep, ultimately leading to time-dependent interfacial delamination under cyclic loading [13]. Therefore, regardless of the constituent material class, the mitigation of localized interfacial shear stresses—whether through functionally graded transitions, topological interlocking [53], or optimized geometric fillets—remains a universal prerequisite for ensuring the long-term dimensional stability of multi-material NTE metamaterials.

Another major limitation is the lack of systematic studies on thermal fatigue and long-term durability. Most existing work focuses on single cycles or short-term thermal loads, neglecting the degradation mechanisms that may arise under repeated thermal cycles. This is particularly relevant for applications in the aerospace and electronics industries, where components are subjected to thousands of thermal cycles over their service life. Furthermore, the trade-off between thermal expansion, stiffness, and manufacturability remains unresolved [26]. High-performance negative thermal expansion (NTE) metamaterials, particularly bimetallic and multi-material lattice architectures, often rely on compliant deformation mechanisms such as bending or rotational hinging. While these mechanisms enable large and tunable negative thermal expansion, they can introduce challenges in structural stiffness and robustness, as well as increased fabrication complexity associated with multi-material integration and interfacial control. Conversely, mechanically optimized lattice designs that prioritize stiffness and load-bearing efficiency typically exhibit reduced NTE magnitudes due to constrained deformation modes. These competing requirements highlight an inherent design-dependent trade-off in NTE metamaterial development.

To explicitly quantify the simulation-to-reality gap discussed in previous sections, Table 5 consolidates the thermal expansion performance of representative engineered NTE metamaterial architectures from the literature. By aligning analytical predictions and physical experimental measurements side-by-side, a systematic discrepancy becomes immediately apparent. In standard macro-scale additive manufacturing, idealized analytical models frequently overestimate the magnitude of negative thermal expansion achievable in physical prototypes. This reduction is primarily driven by process-induced geometric defects, unconstrained localized bulging, and nodal stiffening at joints. As recently demonstrated by Prestes et al. [26], isolated unit cells deviate heavily from predictions, whereas constrained multi-cell matrices align much closer to finite element boundary conditions. Furthermore, when defect-free, ultra-high-resolution manufacturing is employed (e.g., two-photon laser lithography), the predictive gap narrows significantly, underscoring the urgent need for the integration of advanced manufacturing constraints in future design frameworks.

Recent studies further confirm that the current development of negative thermal expansion (NTE) metamaterials is moving from proof-of-concept geometries toward more application-oriented architectures; nevertheless, the literature remains fragmented in terms of materials, length scales, manufacturing routes, and validation protocols. Recent reviews have emphasized the broad potential of NTE metamaterials enabled by additive manufacturing, including polymeric, metallic, ceramic, and multi-material systems, but also highlight that most reported designs are still evaluated under simplified thermal or mechanical boundary conditions [17,18]. In parallel, recent experimental works on metallic and bi-material lattices have demonstrated that the effective CTE can be tuned through geometric design and material pairing; however, these studies also reveal that the measured response is highly sensitive to manufacturing defects, joint stiffness, interfacial quality, and the number of constrained unit cells considered in the experimental configuration [26,91]. Similarly, emerging thermo-stretching-dominated and shape-memory-based architectures have expanded the achievable design space for large and programmable positive or negative thermal deformation, but their long-term dimensional stability, fatigue resistance, and scalability remain insufficiently characterized [27,92]. Therefore, the main research gap identified in this review is the absence of an integrated design–manufacturing–performance framework capable of correlating analytical predictions, numerical models, process-induced defects, interfacial degradation, and long-term thermal cycling behavior across different material classes. The contribution of this review is to provide a comparative and critical synthesis of existing NTE metamaterial architectures, distinguishing between geometry-driven mechanisms, material-driven effects, and manufacturing-induced limitations, while identifying the key requirements that must be addressed to transition NTE metamaterials from laboratory-scale demonstrations to reliable engineering components.

Finally, there is a clear need for integrated design frameworks that combine experimental validation, multiscale modeling, and optimization techniques. The incorporation of data-driven and machine learning approaches offers a promising avenue, but their success depends on the availability of high-quality experimental datasets. Therefore, addressing these challenges will be essential for the advancement of NTE metamaterials, from conceptual designs to reliable engineering solutions.

### Future Research Directions

To fully transition NTE metamaterials from theoretical concepts to deployable engineering solutions, future research must address several critical bottlenecks identified in this review. Foremost, computational frameworks must evolve beyond idealized linear–elastic kinematics. The integration of advanced multiphysics damage models—specifically temperature-dependent Cohesive Zone Modeling (CZM) and viscoplastic creep formulations—is urgently required to accurately predict interfacial delamination, thermal ratcheting, and cyclic thermal fatigue in multi-material systems over extended operational lifespans.

Concurrently, addressing the “simulation-to-reality gap” remains a paramount challenge. Additive manufacturing introduces inherent stochasticity, including micro-porosity, surface roughness, and nodal stiffening. Future research must incorporate these manufacturing realities directly into predictive analytical models and topological optimization loops, shifting from deterministic to probabilistic design frameworks. To navigate the vast, high-dimensional parameter spaces of these stochastic architectures, the accelerated adoption of data-driven inverse design is essential. Generative machine learning frameworks that couple sparse empirical datasets with underlying physical constraints represent the most promising trajectory for discovering novel, high-fidelity NTE geometries while bypassing the computational expense of exhaustive FEA.

**Table 5:** Quantitative comparison of effective Coefficient of Thermal Expansion (CTE) values across representative engineered metamaterials, illustrating the predictive gap between idealized models and physical experimental outcomes.

Architecture & Reference	Constituent Material(s)	Analytical/FEA CTE ($\times 10^{-6}$ K^−1^)	Experimental CTE ($\times 10^{-6}$ K^−1^)	Predictive Discrepancy (Ideal vs. Exp)
Inverted Trapezoid Lattice [13]	Titanium (TA2)/Al (7075-T6)	−76.0 (FEA)	−74.4	Slight Overestimation (∼2%)
I-Square Planar Lattice (NTE) [53]	Al (7075)/Invar	∼−4.0 (Analytical)	−3.26	Analytical Overestimates magnitude by ∼18%
I-Square Planar Lattice (Near-Zero) [53]	Al (7075)/Stainless Steel (431)	∼0.5 (Analytical)	1.59	Significant functional deviation at near-zero threshold
Bi-metallic Triangle Matrix [26]	Invar/Inconel 718	∼−38.0 (FEA)	∼−38.0	Strong match only under metamaterial boundary conditions; isolated cells deviate heavily
3D Chiral Cross Micro-lattice [93]	Polymer (IP-Dip Photoresist)	−50.0 (FEA)	−50.0	Excellent Agreement (Due to ultra-high-resolution fabrication)

Finally, exploring the performance of these architectures under extreme environmental and dynamic loading conditions must be prioritized. As demonstrated by recent advancements in reinforced composites, investigating the kinematic stability of NTE lattices under extreme scenarios—such as blast, shock, and severe thermomechanical cycling—will be critical for their safe deployment in aerospace, defense, and high-performance structural armors.

## 7. Conclusions

Negative thermal expansion (NTE) metamaterials represent a promising class of systems designed to achieve tunable thermal expansion, including near-zero expansion, through geometry-based mechanisms. This review has examined the state of the art from an integrated engineering perspective, covering geometric design, fabrication strategies, and modeling approaches. The analysis reveals that, while significant progress has been made in developing various architectures, from bimetallic lattices to rotational and parametric systems, no single design simultaneously satisfies all performance requirements. Instead, the selection of NTE geometries involves inherent trade-offs between thermal expansion, structural stiffness, manufacturability, and anisotropy.

A crucial finding of this work is the existence of a persistent gap between computational predictions and experimental behavior, due primarily to manufacturing imperfections (particularly process-induced defects in polymer and metal additive manufacturing), material heterogeneity, and the lack of consideration of process-induced effects. Furthermore, the absence of systematic studies on thermal fatigue and long-term durability represents a major limitation for practical applications. To address these challenges, future research should focus on developing multiscale design frameworks that integrate experimental data-driven modeling, multi-objective optimization, and advanced manufacturing techniques. These approaches will be essential for enabling the transition of NTE metamaterials from laboratory-scale demonstrations to robust and scalable engineering solutions.

Bridging the persistent simulation-to-reality gap in NTE metamaterials dictates a fundamental shift in future research priorities. Moving forward, the field must transition from idealized linear–elastic analytical models toward advanced, non-linear multiphysics frameworks. Two critical trajectories are identified. First, for polymeric and multi-material systems, future predictive models must rigorously integrate time-dependent thermoviscoelasticity and cohesive zone formulations to capture interfacial delamination near the glass transition temperature ($T_{g}$). Second, for metallic architectures, computational efforts should prioritize the modeling of elastoplastic evolution, highly localized micro-yielding, and the accumulation of cyclic thermal residual stresses. Targeting these non-linear degradation mechanisms is a mandatory prerequisite for achieving the long-term dimensional stability required for widespread engineering deployment.

## Figures and Tables

**Table 1:** Classification of NTE metamaterials according to dominant deformation mechanism, including representative quantitative performance ranges derived from the consolidated literature to serve as a design selection matrix.

Mechanism	Representative Geometries	Typical CTE ($\times 10^{-6}$ K^−1^)	Normalized Stiffness ($E/E_{s}$)	Relative Density ($\rho^{*}/\rho_{s}$)	Advantages/Limitations
Bending-dominated	Curved bimaterial strips, chiral systems, re-entrant honeycombs	−10 to −100	$10^{-4}$ to $10^{-2}$	0.1 to 0.4	Advantages: broad tunable CTE range and strong thermal actuation. Limitations: lower stiffness and greater sensitivity to thermal fatigue.
Stretching-dominated	Triangular lattices, tetrahedra, octet-truss systems	−5 to −20	$10^{-2}$ to $10^{-1}$	0.1 to 0.3	Advantages: high specific stiffness and mechanical robustness. Limitations: typically narrower CTE tuning range.
Hybrid bending–stretching	Double trapezoidal hexagonal systems, star lattices	−20 to −80	$10^{-3}$ to $10^{-2}$	0.05 to 0.3	Advantages: balanced stiffness and thermal programmability; promising for multifunctional use.
Rotational/hinging-dominated	Connected triangles, anti-chiral lattices, Islamic-pattern systems	−10 to −50	$10^{-3}$ to $10^{-2}$	0.1 to 0.5	Advantages: highly geometry-driven and tunable; often compatible with auxeticity and reversible deployment.
Auxetic + NTE coupled	Re-entrant NPR lattices, L-shaped microstructures, ring–rod	−5 to −40	$10^{-4}$ to $10^{-3}$	0.05 to 0.2	Advantages: combined thermal and mechanical multifunctionality; useful in adaptive systems.
Topology-optimized/distributed compliance	Prescribed CTE continua, graded NTE metamaterials	Target-dependent (e.g., −100 to 0)	$10^{-2}$ to $10^{-1}$	0.3 to 0.6	Advantages: strong design freedom and prescribed global response. Limitations: fabrication complexity.
AI-assisted inverse-designed	Bézier curve systems, deep-autoencoder-designed lattices	Prescribed numerical bounds	Computationally Optimized	Variable	Advantages: efficient exploration of high-dimensional design spaces and simultaneous multiphysics constraints.

**Table 2:** Comparative review of representative negative thermal expansion metamaterials. NPR denotes negative Poisson’s ratio; AM denotes additive manufacturing; FEM denotes finite element method.

Reference	Materials	Fabrication Method	Geometry/Structural Type	Key Features
Dubey et al. (2024) [18]	Aluminum, titanium, and polymers such as PLA and nylon	Additive manufacturing, CNC machining, and assembly	Review of bimaterial strips, chiral systems, re-entrant cells, triangular lattices, tetrahedra, and octahedra	Reported a broad CTE range from highly negative to positive values; classified NTE metamaterials into bending- and stretching-dominated categories.
Zhang and Sun (2024) [36]	Two-constituent multimaterial microstructure	Numerical design approach	L-shaped microstructures; AAAA, AAAC, AACC, and ACAC configurations	Demonstrated coupled auxetic and NTE behavior with tunable two-dimensional and three-dimensional response.
Dubey et al. (2024) [18]	Aluminum, titanium, and polymers such as PLA and nylon	Additive manufacturing, CNC machining, and assembly	Review of bimaterial strips, chiral systems, re-entrant cells, triangular lattices, tetrahedra, and octahedra	Reported a broad CTE range from highly negative to positive values; classified NTE metamaterials into bending- and stretching-dominated categories.
Zhang and Sun (2024) [36]	Two-constituent multimaterial microstructure	Numerical design approach	L-shaped microstructures; AAAA, AAAC, AACC, and ACAC configurations	Demonstrated coupled auxetic and NTE behavior with tunable two-dimensional and three-dimensional response.
Yu et al. (2023) [50]	Generic bimaterial system	Finite element modeling and experimental validation	Re-entrant star-shaped lattice	Achieved near-zero thermal expansion together with broadband vibration suppression.
Xie et al. (DTH system) (2025) [51]	Nylon + PVA	Analytical model + FEA; AM suggested	Double trapezoidal hexagonal lattice	Ultra-lightweight architecture with highly tunable CTE and hybrid bending–stretching behavior.
Yu et al. (2018) [52]	Aluminum + titanium	Laser cutting and mechanical snap-fit assembly	Hourglass-type bimaterial lattice	Exhibited high thermo-mechanical stability, near-zero CTE, and high specific modulus.
Wei et al. (2016, 2018) [53,54]	Al6061 + Invar/Ti6Al4V	Laser cutting, assembly, and metallic multimaterial realization	2D and 3D triangular lattices	Stretching-dominated systems with high specific stiffness and programmable thermal expansion.
Grima and Kiselev (2007, 2024) [55,56]	Materials with contrasting elastic and thermal properties	Analytical design and polymeric prototypes	Triangular grids and layered architectures	Early examples of rotational/auxetic-inspired NTE mechanisms with anisotropic behavior.
Lakes (1996) [57]	Bimaterial strips	Conventional prototyping	Curved bimaterial strips	Foundational mechanical concept for curvature-driven negative thermal expansion.
Wu et al. (2019) [58]	Generic bimaterial system	Polymeric AM and assembly	Anti-chiral lattices	Thermal contraction governed by node rotation and ligament deformation.
Ai and Gao (2017, 2018) [59,60]	Bimaterial system	Numerical simulations with experimental prototypes	Re-entrant honeycombs and 3D star-like cells	Combined NTE with negative Poisson’s ratio in double-negative metamaterials.
Xu et al. (2016, 2018) [61,62]	Al6061 + Ti6Al4V	Laser cutting and assembly	Octet-truss, tetrahedral, and hierarchical lattices	High specific stiffness and programmable thermal expansion through stretching-dominated architecture.
Lim et al. (2019, 2023) [11,15]	Generic bimaterial system	Theoretical analysis	Ring–rod and tetrahedral architectures	Coupled auxetic and NTE behavior, including negative volumetric thermal expansion.
Li et al. (2018) [63]	Bimaterial system	Simulation and prototyping	Hoberman-sphere-inspired architecture	Thermally controlled radial expansion with geometrically tunable effective CTE.
Ndlovu et al. (2009) [64]	High-CTE internal members + low-CTE frame	Beam-theory-based analytical approach	Hexagonal cellular grid	Provided analytical solutions for temperature-dependent thermal expansion in cellular systems.

**Table 3:** Design–fabrication–application mapping of representative NTE systems.

Structural Concept	Typical Fabrication Route	Potential Applications
Bimaterial curved strips/bilayers	Conventional lamination, layer bonding, laser cutting	Thermal actuators, thermostatic systems, precision compensators.
Rotational and auxetic lattices	Polymeric AM, metallic AM, laser-cut assembly	Adaptive panels, deployable systems, thermally stable lightweight structures.
Trapezoidal and star-like metamaterials	Multimaterial AM, FEM-guided prototyping	Thermal management panels, multifunctional structural components.
Bézier curve and ML-designed architectures	Inverse design + additive manufacturing	Aerospace thermal compensation, precision engineering, programmable deformation systems.
MEMS-scale lattices	Micro-fabrication, lithography, thin-film processing	Microactuators, thermal microsensors, precision microsystems.
NiTi-based active metamaterials	Laser powder bed fusion, metallic AM	Thermally adaptive devices, smart actuators, active load-bearing systems.
MOFs and intrinsic NTE frameworks	Chemical synthesis, crystallographic processing	Thermal compensation fillers, hybrid composites, functional porous materials.
Topology-optimized graded systems	Computational design + advanced manufacturing	Functionally graded thermal control devices and customized thermal expansion interfaces.

**Table 4:** Glass transition temperatures ($T_{g}$) of common AM polymers and their effective operational envelopes for reliable NTE kinematic performance.

Polymer	Typical AM Process	Approx. $T_{g}$ (°C)	Max Safe NTE Temp (°C)
Polylactic Acid (PLA) [85]	FFF	60–65	45
Polyethylene Terephthalate Glycol (PETG) [86,86]	FFF	80–85	65
Acrylonitrile Butadiene Styrene (ABS) [87]	FFF/SLA	105–110	90
Polyamide 12 (Nylon) [88]	LPBF/FFF	45–50 *	40
Thermoplastic Polyurethane (TPU) [89,90]	FFF/SLS	−50 to −20 **	Varies (Rubbery State)

* Highly dependent on environmental moisture content and crystallinity. ** Refers to the soft-segment $T_{g}$; TPU operates natively in its elastomeric rubbery plateau at room temperature.

## Data Availability

The original contributions presented in this study are included in the article. Further inquiries can be directed to the corresponding author.
